# Rerouting glucose metabolism of therapeutic T-cells for cancer: live longer, perform better

**DOI:** 10.1097/IN9.0000000000000063

**Published:** 2025-07-05

**Authors:** Zhongyi Dong, Jianmei W. Leavenworth

**Affiliations:** 1 Department of Neurosurgery, University of Alabama at Birmingham, Birmingham, AL, USA; 2 Graduate Biomedical Sciences Program, University of Alabama at Birmingham, Birmingham, AL, USA; 3 Department of Microbiology, University of Alabama at Birmingham, Birmingham, AL, USA; 4 The O’Neal Comprehensive Cancer Center, University of Alabama at Birmingham, Birmingham, AL, USA

**Keywords:** adoptive cell therapies, pharmacologic metabolic rewiring, pyruvate dehydrogenase kinase 1, dichloroacetate, epigenetic reprogramming, exhaustion, immune memory

## Abstract

A significant barrier to the success of adoptive cell therapies (ACTs) in cancer treatment is the inadequate persistence of T-cells following infusion. In vitro T-cell expansion is a crucial component of ACTs; therefore, preconditioning during culture may enhance their in vivo survival and therapeutic efficacy. Here, we discuss a recent article by Greg Delgoffe and colleagues that was published in *Cell Metabolism* in April 2025, providing evidence that pharmacologic metabolic rewiring of activated T-cells during in vitro expansion enhances their engraftment postinfusion and improves cellular immunotherapies.

T-cell-based adoptive cell therapies (ACTs) have demonstrated potential in cancer treatment, though clinical responses remain suboptimal ^[1–3]^. This approach involves the administration of tumor-reactive T-cells, which are isolated and expanded ex vivo from a patient’ tumor or peripheral blood. These cells can be further genetically engineered to express tumor-specific receptors, such as chimeric antigen receptors (CARs) in CAR-T therapy or T-cell receptors (TCRs) in TCR-T therapy, thereby enhancing their antigen specificity and antitumor efficacy. A major challenge facing all ACTs is the limited persistence and survival of T-cells following infusion. These therapies rely on an in vitro T-cell expansion phase, during which cells are exposed to supraphysiologic levels of glucose, mitogens, cytokines, and other nutrients. However, T-cells expanded in vitro develop a markedly different metabolic profile compared with those expanded in vivo ^[4]^. Frisch et al ^[5]^ further explored this metabolic divergence and proposed that pharmacologically redirecting glycose flux towards mitochondrial metabolism during in vitro T-cell expansion could enhance their in vivo persistence and, in turn, improve the efficacy of ACT in cancer treatment.

Using TCR transgenic CD8^+^ T-cells, Frisch et al first demonstrated that after adoptive transfer into mice, the expanded T-cells exhibited a significantly enhanced mitochondrial profile and depended on oxidative metabolism. In contrast, in vitro-expanded T-cells primarily engaged in excessive aerobic glycolysis, as shown by oxygen consumption rate, extracellular acidification rate, and other metabolic measurements. Early TCR activation triggers rapid aerobic glycolysis by phosphorylating and activating pyruvate dehydrogenase kinase 1 (PDHK1), which supports effector T-cell function. PDHK1 inhibits the entry of pyruvate into mitochondria by phosphorylating pyruvate dehydrogenase, thereby diverting pyruvate toward lactate production instead ^[6]^. Since dichloroacetate (DCA), a small-molecule compound, inhibits PDHK1, the authors treated activated CD8^+^ T-cells with DCA during the expansion phase but not during initial activation to reduce excessive aerobic glycolysis while preserving their capacity for proliferation and expansion. This shift in glucose flux toward the mitochondria enhanced mitochondrial capacity, increased adenosine triphosphate (ATP) production, and promoted the conversion of glucose into mitochondrial metabolites such as citrate, fumarate, and malate while reducing dependence on glycolysis in both murine and human peripheral blood mononuclear cell (PBMC)-derived CD8^+^ T-cells. Importantly, these DCA preconditioned CD8^+^ T-cells achieved superior tumor clearance and significantly extended survival in mice bearing B16 melanoma. This metabolic reprogramming also enhanced the therapeutic efficacy of human PBMC-derived CAR-T-cells in immunodeficient mice bearing subcutaneous human tumor xenografts. Notably, these T-cells elicited robust immune memory, allowing the mice to effectively control B16 melanoma upon rechallenge. Interestingly, DCA-treated T-cells did not show significant functional or phenotypic changes in the tumor 7 days posttransfer but rather demonstrated better survival and engraftment. Upon infusion, DCA-treated T-cells were found in higher numbers across the blood, spleen, lymph nodes, and tumors. Remarkably, these cells remained detectable in the circulation 300 days postinfusion, suggesting that DCA-induced metabolic reprogramming supports long-term engraftment of transferred T-cells. This approach may help overcome one of the major limitations of ACTs: poor persistence following infusion.

Frisch et al further investigated the mechanisms by which DCA conditioning enhanced T-cell engraftment into tumor-bearing mice. The tumor microenvironment (TME) is known to suppress mitochondrial metabolism in T-cells, leading to their exhaustion ^[7]^. In contrast, DCA-treated T-cells produced less lactate and more efficiently utilized physiological carbon sources such as lactate, pyruvate, and glutamine, thereby enhancing their metabolic adaptability and survival. Detailed profiling revealed that these cells exhibited increased stemness characterized by upregulation of SLAMF6 and TCF-1–along with greater central memory formation and a reduced exhaustion phenotype. Notably, the increase in memory formation was less evident in human T-cells. T-cell metabolic pathways produce substrates essential for the activity of epigenetic enzymes, thereby linking cellular metabolism to epigenetic regulation ^[8]^. Among the various epigenetic modifications, histone acylation such as acetylation and lactylation, which involve the addition of acyl groups to lysine residues on histones has been shown to regulate gene transcription in CD8^+^ T-cells and influence their function ^[9]^. Alongside the reduction in histone lactylation caused by DCA treatment, increased levels of histone modifications H3K27Ac and H3K9Ac were observed in these conditioned T-cells. Importantly, these changes were not widespread across the genome but were instead enriched at specific loci associated with key stemness genes. This epigenetic reprogramming was driven, at least in part, by enhanced mitochondrial export of citrate into the cytosol, leading to increased nuclear acetyl-CoA levels following DCA conditioning (Figure 1).

**Figure 1.**
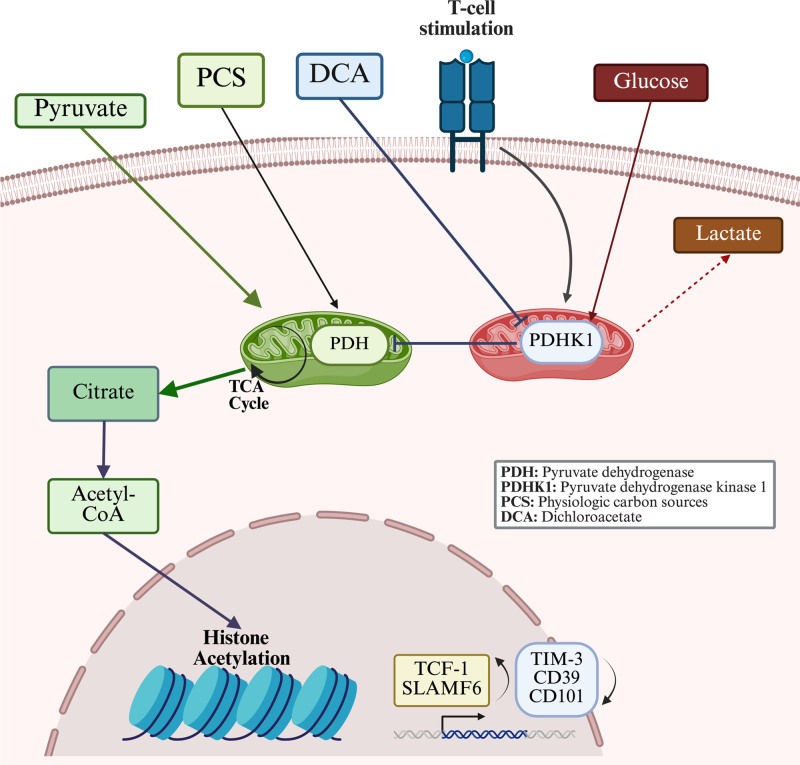
**Rerouting glucose flux into mitochondria by DCA to increase T-cell longevity.** DCA preconditioning of T-cells during in vitro expansion redirects glycolysis towards mitochondrial metabolism, reducing lactate production and promoting the utilization of physiological carbon sources. This shift enhances citrate export from the mitochondria to the cytosol, leading to increased nuclear acetyl-CoA levels, which, in turn, drive histone modifications and epigenetic remodeling at stemness-associated gene loci, supporting improved persistence after in vivo infusion. CoA, coenzyme A; DCA, dichloroacetate. This figure was created using Biorender.

The study by Frisch et al has further deepened our understanding of how metabolic reprogramming linked to epigenetic remodeling impacts T-cell life span and consequently antitumor outcomes following infusion. Several questions arising from this study could be explored further. First, the reversibility of DCA-mediated inhibition postinfusion appeared critical, as continued DCA administration abolished the improved antitumor efficacy; however, T-cells were able to resume glycolytic function once DCA was removed from the culture. Beginning 13 hours postinfusion, a greater proportion of DCA-conditioned T-cells compared to untreated T-cells were detected in circulation, indicating a more effective transition from in vitro to in vivo. Nonetheless, it remains uncertain whether these cells retain their metabolic advantages and epigenetic remodeling within the TME, or how long such imprinting persists. Second, the in vivo persistence and engraftment of DCA-conditioned T-cells occurred independently of antigen availability. It would be interesting to explore how the metabolic advantages conferred by DCA conditioning might counteract exhaustion caused by chronic TCR stimulation within the TME, especially in immunodeficient settings lacking CD4^+^ T-cell help ^[10]^. Third, while histone acylation can influence a broad range of genes, DCA-induced epigenetic reprogramming appeared to be focused on specific gene loci. This raises the question of whether the effect is subset-specific or context-dependent. Fourth, the effects of DCA conditioning were primarily investigated in CD8^+^ T-cells. It remains to be seen whether this pharmacologic metabolic rewiring is also effective in other cell types, such as CD4^+^ T-cells, and in additional solid tumor models beyond subcutaneous settings. Finally, enhanced persistence and survival of the stem-like T-cell population were central to the improved tumor control following in vitro DCA treatment. Since anti-PD-1 promotes differentiation of these stem-like cells into effector subsets ^[11,12]^, combining DCA-conditioned T-cell infusion with anti-PD-1 checkpoint blockade may yield synergistic antitumor effects—a promising therapeutic approach warranting further investigation.

In summary, this study provides proof-of-concept that metabolic rewiring of T-cells during in vitro expansion enhances their longevity postinfusion, ultimately improving antitumor efficacy. Future clinical studies are needed to assess the safety and efficacy of this approach in cancer patients. Moreover, combining it with other metabolic modulators, immune checkpoint inhibitors, or cytokine therapies offers promising avenues to further enhance cancer immunotherapy.

## Conflict of interest

The authors declare that they have no conflicts of interest.

## Funding

J.W.L. is supported by NIH R01AI148711, R01AI148711-03S1/04S1 (via NIA), R01CA276190, R21CA278853, DoD HT9425-23-1-0792, American Cancer Society RSG-23-1038722-01-IBCD, O’Neal Comprehensive Cancer Center Pre-R01, and the UAB faculty start-up funds. No funding was used to produce this commentary.

## Acknowledgments

The figure was created with BioRender.com.
